# Syndecan 4 Is Involved in Mediating HCV Entry through Interaction with Lipoviral Particle-Associated Apolipoprotein E

**DOI:** 10.1371/journal.pone.0095550

**Published:** 2014-04-21

**Authors:** Mathieu Lefèvre, Daniel J. Felmlee, Marie Parnot, Thomas F. Baumert, Catherine Schuster

**Affiliations:** 1 Inserm, U1110, Research Institute on Viral and Hepatic Disease, Strasbourg, France; 2 Université de Strasbourg, Strasbourg, France; 3 Pôle hépato-digestif, Hôpitaux Universitaires de Strasbourg, Strasbourg, France; Saint Louis University, United States of America

## Abstract

Hepatitis C virus (HCV) is a major cause of liver disease worldwide and HCV infection represents a major health problem. HCV associates with host lipoproteins forming host/viral hybrid complexes termed lipoviral particles. Apolipoprotein E (apoE) is a lipoprotein component that interacts with heparan sulfate proteoglycans (HSPG) to mediate hepatic lipoprotein uptake, and may likewise mediate HCV entry. We sought to define the functional regions of apoE with an aim to identify critical apoE binding partners involved in HCV infection. Using adenoviral vectors and siRNA to modulate apoE expression we show a direct correlation of apoE expression and HCV infectivity, whereas no correlation exists with viral protein expression. Mutating the HSPG binding domain (HSPG-BD) of apoE revealed key residues that are critical for mediating HCV infection. Furthermore, a novel synthetic peptide that mimics apoE’s HSPG-BD directly and competitively inhibits HCV infection. Genetic knockdown of the HSPG proteins syndecan (SDC) 1 and 4 revealed that SDC4 principally mediates HCV entry. Our data demonstrate that HCV uses apoE-SDC4 interactions to enter hepatoma cells and establish infection. Targeting apoE-SDC interactions could be an alternative strategy for blocking HCV entry, a critical step in maintaining chronic HCV infection.

## Introduction

Hepatitis C virus (HCV) infection is a major worldwide cause of liver disease, including liver cirrhosis and hepatocellular carcinoma [1]. The current standard of care treatment is composed of pegylated interferon alpha, ribavirin, and one of the newly developed direct-acting antiviral (DAA) agents, telapravir or boceprevir, but this regimen is limited by prohibitively high costs, resistance mutations, and unwanted side effects [2], [3]. One hallmark of HCV is its unique association with host lipoproteins including very-low density lipoproteins (VLDL), in host/viral hybrid complexes termed lipoviral particles. HCV relies on elements of VLDL assembly for viral production, and the virion is associated with the apolipoproteins (apo), apoE, apoB, apoAI, and apoCI [4], [5], [6]. Evidence indicates that HCV utilizes aspects of lipoprotein metabolism as a mechanism of hepatocyte egress and in the early steps of infection [7].

**Figure 1:**
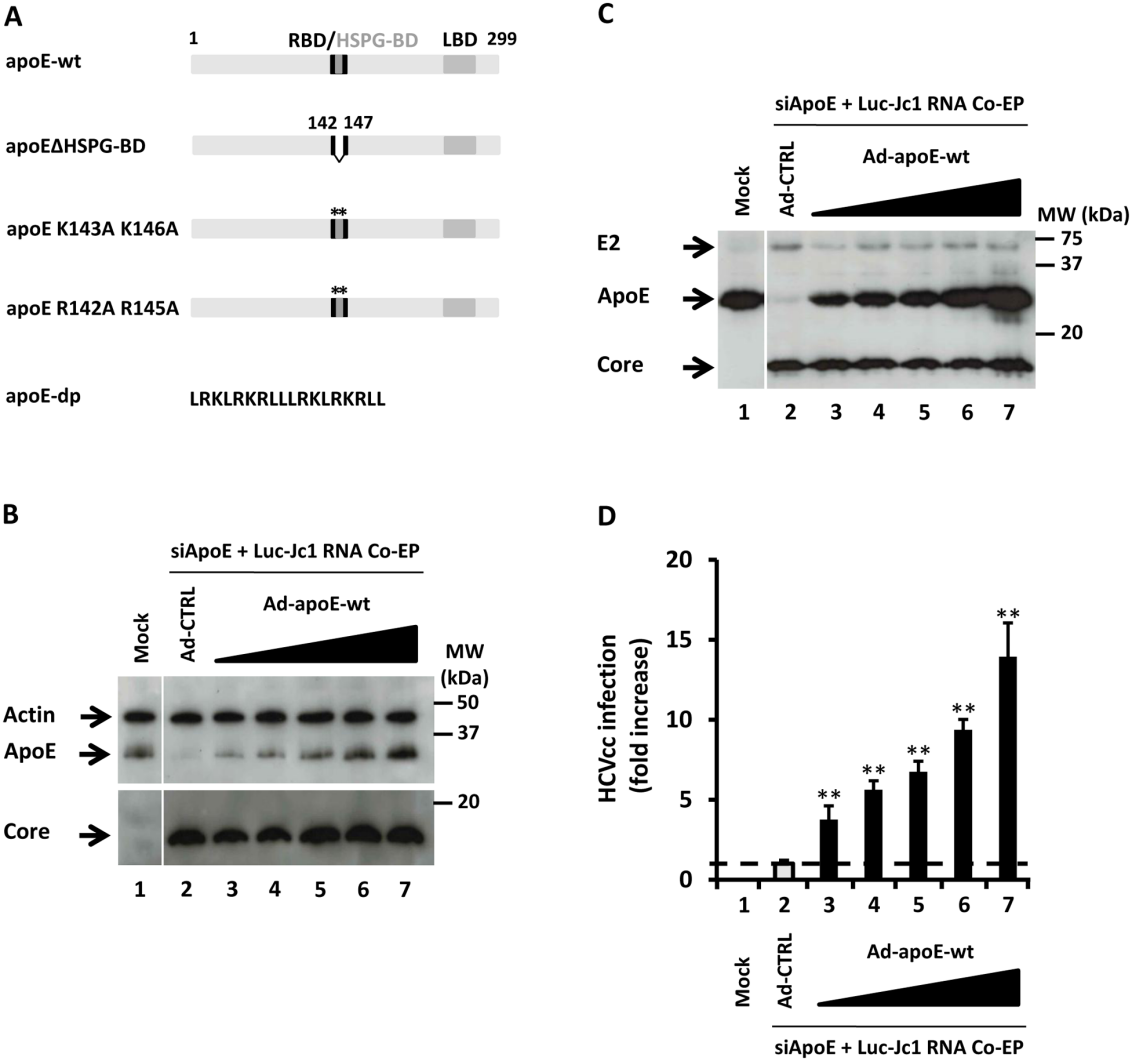
Ectopic expression of apoE dose-dependently stimulates HCV production. (A) Schematic of apoE mutants and apoE-derived peptide sequence. Receptor binding domain (RBD: amino acids 136–150) and heparan sulfate proteoglycan binding domain (HSPG-BD: amino acids 142–147) are represented. Mutations of the apoE HSPG-BD (apoEΔHSPG-BD, apoE K143A, K146A, and apoE R142A, R145A) were generated by site-directed mutagenesis. (B) Huh7.5.1 cells were either co-electroporated (Co-EP) with luciferase-encoding HCV RNA (Luc-Jc1) and siRNA targeting endogenous apoE expression (siApoE) (2–7) or mock-transfected (1). 24 h post-transfection, cells were transduced with adenoviruses expressing GFP (Ad-CTRL) as a control, or with increasing concentrations of adenoviruses expressing wt apoE (Ad-apoE-wt), representing 1∶100–1∶5 dilutions, and numbered from 2 to 7 according to increasing concentration. Three days post-transduction, intracellular apoE, actin and HCV core expression was determined by immunoblot of cell lysates. (C) Extracellular culture supernatants of the cells from (B) with corresponding number designations were concentrated by sucrose cushion. ApoE, HCV E2, and core expression were tested by Western blot. (D) HCV infection from apoE modulated cells was conducted by exposing naïve Huh7.5.1 cells to culture media from cells transfected with HCV RNA and transduced with increasing concentrations of Ad-apoE-wt or with Ad-CTRL with number designations corresponding to (B) and (D). 3d post-infection, infectivity was measured by luciferase reporter activity. HCVcc infection is expressed as a percentage relative to apoE-silenced cells transduced with Ad-CTRL. Results are expressed as mean±SD of the experiment performed in triplicate (** = *P*<0.001).

ApoE is a VLDL component that plays a key role in the HCV life cycle. We have previously shown that apoE interacts with the HCV NS5A protein and is critical for HCV assembly and release [8], while others have demonstrated a role for apoE in HCV entry [9], [10]. It has been demonstrated that apoE interacts with heparan sulfate proteogycans HSPG [10] and the low-density lipoprotein receptor (LDL-R) [11] during HCV hepatocyte entry. However, the role of LDL-R is disputed [12], and identifying the key HSPG family member involved during HCV entry remains unclear. ApoE is a 299 amino acid protein that has an N-terminal receptor-binding domain (RBD) and a C-terminal lipid-binding domain (LBD) (Fig. 1A). Within the RBD, there is a small region enriched in positively charged amino acids that interacts with heparan sulfate proteoglycans (HSPG) on the cell surface. Evidence indicates that this interaction is an important first step in hepatic clearance of VLDL remnant lipoproteins, particularly with a subset of HSPGs, syndecans (SDCs). SDCs consist of core transmembrane proteins with negatively charged heparan sulfate side chains post-translationally added to the extracellular moiety. Four SDCs (1 through 4) are found in humans. SDC1 is highly expressed in epithelial cells such as hepatocytes, SDC2 in endothelia and fibroblasts, and SDC3 in neuronal tissues, while SDC4 is predominantly co-expressed with other SDCs. It has been shown that the SDC family is involved in wound healing and tumor progression [13]. Moreover, SDC1 is the primary proteoglycan receptor mediating binding, uptake, and degradation of VLDL both *in vitro* [14] and *in vivo* [15], [16]. Since HCV utilizes aspects of lipoprotein metabolism and apoE is on the virion surface, we aimed to determine the apoE-interacting host factor that mediates HCV infection on the hepatocyte surface.

## Experimental Procedures

### Cell Lines, Plasmids and Reagents

HEK293T and Huh7.5.1 cells were cultured as described [17]. Plasmid pFK-Luc-Jc1 (Luc-Jc1) and pFK-Jc1 (Jc1) constructs have been previously described [18], [19], [20]. HA-SDC4 and HA-SDC4-Y180L plasmids were kind gifts from Martin J. Humphries, University of Manchester [21]. Human apoE-encoding cDNA was obtained from OriGen USA (Rockville, MD, USA). An apoE-derived di-peptide (apoE-dp) corresponding to apoE region 141–149 (LRKLRKRLLLRKLRKRLL) was synthesized (Inserm U977 Strasbourg, France), and purified by high pressure liquid chromatography (HPLC) to 95% purity. The peptide contains N-terminal acetyl and C-terminal amide capping groups. Control peptide (CYEKFKTPKDKTKM) was synthesized by ProteoGenix SAS (Schiltigheim, France) and purified by HPLC to 82% purity. Heparin (H3393) was obtained from Sigma-Aldrich (Sigma-Aldrich, St Louis, USA). Human VLDL (BT-909) was purchased from Biomedical Technologies Inc. (Stoughton, MA 02072 USA).

### Antibodies

Mouse monoclonal anti-apoE (ab8226) and mouse monoclonal anti-beta actin (ab1906) antibodies were obtained from Abcam (Paris, France). Mouse monoclonal anti-HA-tag (clone F-7, sc-7392) antibody was obtained from Santa Cruz Biotechnology, Inc. Anti-mouse IgG coupled to horseradish peroxidase (HRP) (NXA931) and ECL Western blotting detection reagents (RPN2106) were obtained from GE Healthcare.

### RNA Interference Assay

Specific siRNA targeting endogenous apoE 3′ UTR (siApoE) (5′ CUGCAGCGGGAGACCCUGU 3′), specific siRNA targeting syndecan-1 (siSDC1, L-010621), CD81 (siCD81, L-017257-00-005), syndecan-4 (siSDC4, M-003706-01), or each siSDC4 aliquoted individually (siSDC4-1 to siSDC4-4, J-003706-07 to J-003706-10 respectively), and scrambled control siRNAs (siCTRL, DY-547) were purchased from Dharmacon (Dharmacon Inc., Chicago, IL USA). siRNAs were transfected using Lipofectamine RNAiMAX transfection protocol purchased from Life technologies, or electroporated as described previously [8]. Three days post-transfection, target gene expression was tested by quantitative RT-PCR using TaqMan Gene Expression Assay (SDC1: PN4453320, SDC4: PN4448892) purchased from Life technologies or by Western blot as described previously [8].

### Recombinant Adenoviruses

The recombinant adenoviral genomes were generated as infectious plasmids by homologous recombination in *E. coli*, as described previously [22]. Briefly, a PCR-amplified fragment encoding siApoE-resistant cDNA, HA-SDC4 wt or HA-SDC4 Y180L was inserted into the adenoviral shuttle plasmid pTG13387 (US patent 2002/0019051 A1). In the resulting vector, cDNAs are under the control of a cytomegalovirus promoter, and their sequences are surrounded by adenoviral sequences (nt 1 to 458 and nt 3328 to 5788 of the adenovirus type 5 (Ad5) genome). All apoE encoding inserts were siApoE resistant and were obtained by site-directed mutagenesis. The cDNAs encoding shuttle plasmids obtained were used for homologous recombination with adenoviral sequences of the backbone vector pTG6624 [22]. The resulting adenoviral plasmids contain the full-length adenoviral genome with a deletion in E3 (nt 28592 to 30470). The E1 region (nt 459 to 3327) is replaced with the sequence encoding apoE-wt, apoE-mutant, HA-SDC4-wt, or HA-SDC4-Y180L. Recombinant adenoviruses Ad-apoE-wt, Ad-apoE-mutant, Ad-HA-SDC4-wt, or Ad-HA-SDC4-Y180L were generated by transfection of these plasmids into the 293T packaging cell line after *Pac*I digestion. Ad-CTRL, which was used as a control, is a recombinant adenovirus encoding green fluorescent protein (GFP) [23]. Preparation of adenoviruses was previously described [24].

### HCV Production and Infectivity

Luc-Jc1 or Jc1 HCV RNA was obtained by T7 *in vitro* transcription of plasmid pFK-Luc-Jc1 or pFK-Jc1, respectively. Huh7.5.1 cells were co-electroporated with Luc-Jc1 RNA and siApoE or siCTRL, as described previously [8], to obtain cell-culture derived HCV particles (HCVcc). The next day, cells were transduced with adenoviruses expressing apoE-wt, apoE-mutants, or GFP as a control. Three days post-transduction, HCV replication was assessed using luciferase assay of the cell lysates as previously described [8]. Cell supernatants were collected and infectivity was quantified by luciferase assay, 3d post-infection of naïve Huh7.5.1 cells [8]. The presence of HCVcc in the cell supernatants was verified following sucrose cushion virion concentration, as previously described [8]. Briefly, HCVcc were purified from cell culture supernatant on a 20% sucrose cushion by ultracentrifugation using a SW 55 Ti rotor (Beckman Coulter, Inc.) at 30,000 rpm for 4 h at 4°C. The viral pellet was resuspended in lysis buffer. Levels of apoE, HCV glycoprotein E2, and HCV core protein were compared by Western blot.

### Analysis of HCVcc Binding

Huh7.5.1 cells were incubated with Luc-Jc1 HCVcc at a multiplicity of infection (MOI) of one for 1 h at 4°C. After thorough washing with PBS, HCVcc binding was analyzed immediately by qRT-PCR [17], or HCVcc infection was measured 48 h later by luciferase assay [8]. VLDL competition was conducted by mixing Luc-Jc1 HCVcc particles with VLDL at serial dilutions prior to incubation on naïve Huh7.5.1 cells for 2 h at 4°C. After three washes with PBS, HCVcc binding was analyzed immediately by quantification for viral RNA using qRT-PCR [17].

#### HCV pseudoparticle (HCVpp) production and assay

Luciferase-encoding HCVpp strain J6 were produced as previously described [17]. HCVpp infection was performed using the same protocol as HCVcc infection above. Briefly, 3 days after siRNA transfection, transfected-cells were infected with HCVpp for 4 h at 37°C. Three days post-infection, the cells were lysed and HCVpp entry was analyzed using luciferase assay.

### Syndecan 4 Complementation Assay

Huh7.5.1 cells were transfected with oligonucleotides to knockdown expression of SDC4 (siSDC4) or a siRNA control (siCTRL). 24 h post-transfection, cells were transduced with adenoviruses expressing either GFP (Ad-CTRL) as a control, SDC4-wt, or SDC4-Y180L. Three days post-transduction, tranfected and transduced cells were infected with Luc-Jc1 HCVcc for 4 h at 37°C. Three days post-infection, infectivity was measured by luciferase reporter activity. HCVcc infection is expressed as a percentage relative to siCTRL-silenced cells transduced with Ad-CTRL.

## Results

### Modulation of apoE Expression Regulates HCV Infectivity

To identify apoE binding partners involved in the HCV life cycle, we established a functional trans-complementation assay to replace endogenous apoE expression with ectopic expression of wild-type or mutated apoE. Coupling this system with HCVcc expression allowed mapping of apoE functional domains that are important for HCV infection and production (Fig. 1A). Endogenous apoE was silenced by siRNA (siApoE) confirmed by Western blot (Fig. 1B) in HCV transfected cells, then trans-complemented by increasing concentrations of apoE via adenoviral transduction lacking sequences prone to siApoE (Fig. 1B). Intracellular apoE levels increased in a dose-dependent manner depending on Ad-apoE-wt concentration (Fig. 1B) whereas HCV core protein quantities were not altered indicating that apoE does not alter expression or stability of this protein (Fig. 1B). To determine how altering apoE expression affects apoE and HCV production, extracellular components were concentrated on a sucrose cushion. While unmanipulated Huh7.5.1 produce basal levels of apoE, modulation and trans-complementation can diminish or stimulate this production (Fig. 1C). This change was in contrast to HCV glycoprotein E2 and core levels produced, which were unaffected by altered apoE expression (Fig. 1C). Interestingly, HCV infectivity correlated strongly with apoE expression levels without reaching saturation (Fig. 1D), while there was no such correlation with viral proteins. These results clearly indicate the key role of apoE expression in assembly and production of infectious HCV particles.

### The HSPG Binding Domain of apoE is Critical for HCV Entry

**Figure 2:**
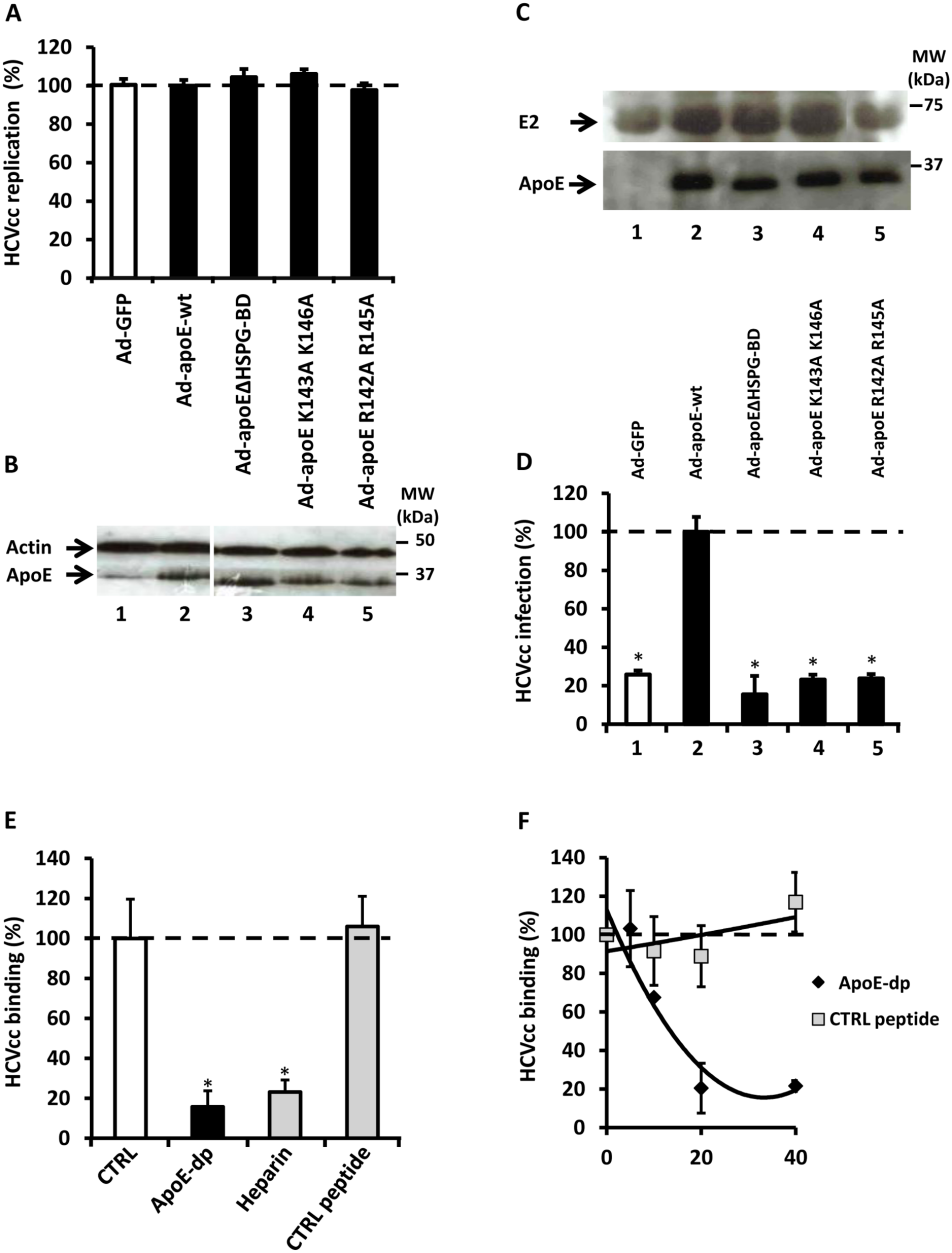
ApoE HSPG-BD is required for HCVcc infectivity. (A–D) Huh 7.5.1 cells were co-electroporated with luciferase-encoding HCV RNA (Luc-Jc1) and siRNA targeting endogenous apoE expression. 24 h post-transfection, cells were transduced with adenoviruses expressing either GFP (Ad-CTRL) as a control, apoE-wt (Ad-apoE-wt), or apoE-mutants (Ad-apoEΔHSPG-BD, Ad-apoE K143A K146A, and Ad-apoE R142A R145A). 3d post-transduction, culture media from cells was harvested and components were concentrated by ultrafiltration and sucrose cushion. (A) HCV replication in transfected and transduced cells was monitored by measuring luciferase activity. (B) Comparable wt or mutated-apoE intracellular expression in adenoviral transduced HCV replicating cells was confirmed by Western blot with lanes corresponding to those of Fig. 2A. (C) Concentrated culture supernatants were tested for HCV glycoprotein E2 and apoE by Western blot, representative of three independent experiments. (D) Infectivity of HCVcc generated from transduced cells was monitored by exposure of naïve Huh 7.5.1 cells to cell culture supernatants, and assaying luciferase activity 3d post-infection. HCVcc infection is expressed as percentage relative to wt apoE trans-complemented cells. Results are expressed as mean±SD of the experiment performed in triplicate (* = *P*<0.005). Lanes correspond to those of Fig. 2C. (E) Synthesized peptide corresponding to apoE HSPG-BD (apoE-dp) (20 µg/mL), heparin (20 µg/mL) or a control peptide (CTRL peptide) were pre-incubated for 1 h with Luc-Jc1 HCVcc prior to addition to the cells for 1 h at 37°C. Cells were washed three times with PBS and media was replaced. Infectivity was assayed by luciferase activity of cell lysates 2d post-infection HCVcc attachment is expressed as a percentage relative to mock-treated control. (F) Serial increasing concentrations of apoE-dp were pre-incubated for 1 h with Luc-Jc1 HCVcc (MOI = 1) prior to 1 h incubation with Huh 7.5.1 cells at 4°C to allow attachment. Subsequently, cells were washed three times with PBS and media was replaced. Infectivity was assayed by luciferase activity of cell lysates 2 days post-infection. HCVcc attachment is expressed as a percentage relative to mock-treated control. Results are expressed as mean±SD of three independent experiments performed in triplicate. (* = p<0.005).

Since we show that apoE plays a central role in HCV infection, we endeavored to map regions within apoE that mediate this function. It is known that a key role of apoE is to mediate TRL remnant uptake by hepatocytes through binding HSPG via a positively charged region of the protein termed HSPG binding domain (HSPG-BD). With the concept that HCV may co-opt this entry pathway, we made adenoviral vectored apoE mutants with either deleted HSPG-BD (Ad-apoEΔHSPG-BD), or two mutants with cationic residues lysines or arginines replaced by alanines (apoE_K143A,K146A_ or apoE_R142A,R145A_) (Fig. 1A). The introduction of these mutations did not affect intracellular transfected HCV RNA levels relative to uncomplemented apoE knockdown or apoE-wt, revealing that apoE modulation does not alter HCV replication (Fig. 2A). Western blot analysis determined that apoE expression of the mutants was comparable to wt, indicating that the mutants were stable (Fig. 2B). By concentrating culture media components of the cells by ultracentrifugation on a sucrose cushion, we determined that despite robust apoE production from all the mutants, revealed by Western blot (Fig. 2C), only apoE-wt retained the capacity to mediate HCV infection (Fig. 2D). These conclusive results demonstrate that HCV infection is dependent on the positively charged residues within the HSPG-BD of apoE.

Since these results indicate that apoE mediates infection through basic residues contained in the HSPG-BD, we generated an apoE derived peptide (apoE-dp) consisting of a repeated sequence of the HSPG-BD (Fig. 1A). We aimed to define that HSPG-BD actively mediates HCV infection, and to rule out conformational changes that may result from introducing mutations. As expected, apoE-dp was capable of out-competing HCV binding on the surface of hepatoma cells to a similar degree as the HCV entry inhibitor heparin (Fig. 2E) [25], whereas control peptide (CTRL peptide) had no effect on HCV infection. Through inhibiting binding, apoE-dp likewise inhibited HCV infection in a dose-dependent manner (Fig. 2F).

### Syndecan 4 Mediates HCV Entry

**Figure 3:**
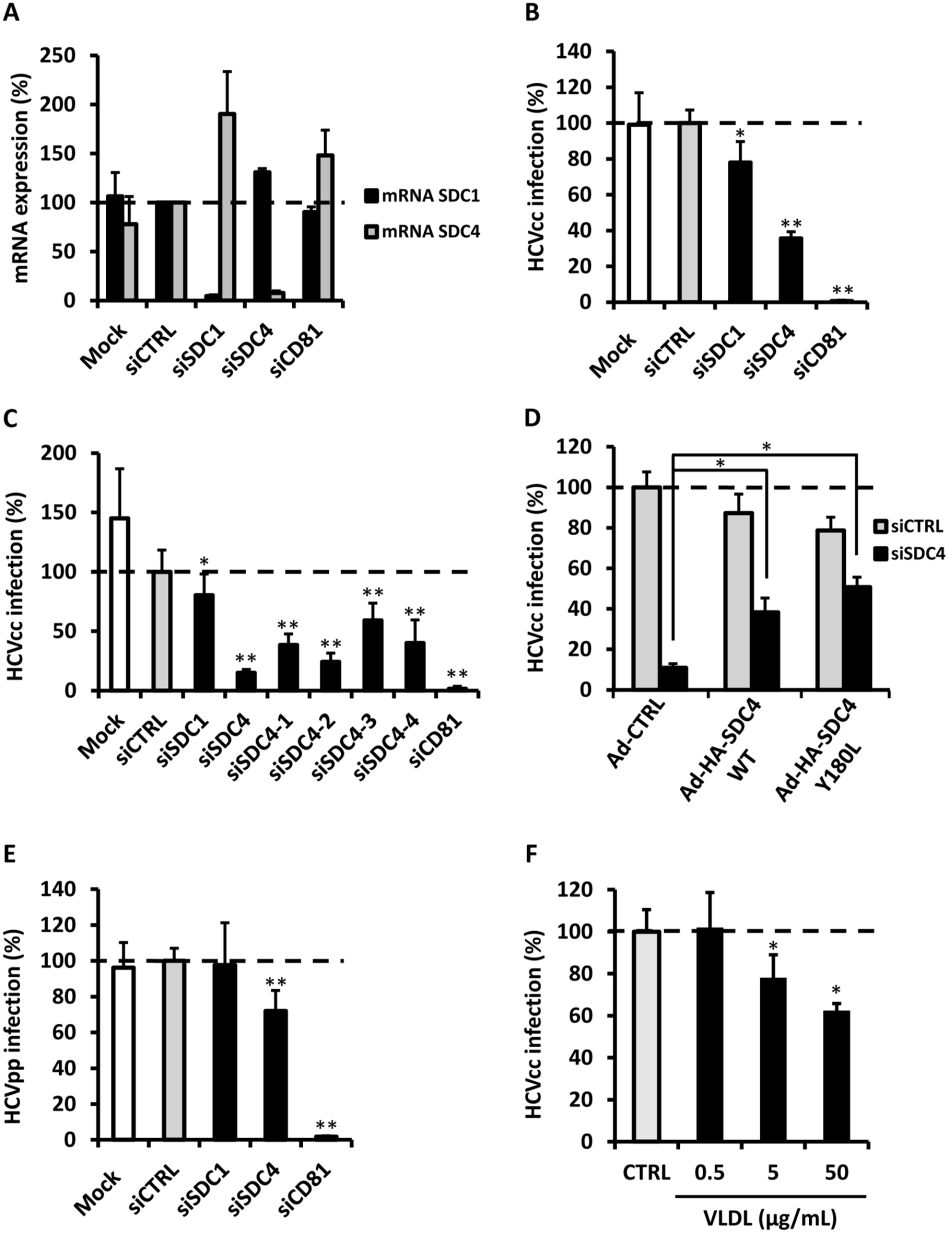
Syndecan 4 is involved in HCV infection. (A) Huh 7.5.1 cells were transfected with oligonucleotides to knockdown expression of SDC1 (siSDC1), SDC4 (siSDC4), CD81 (siCD81) or a siRNA control (siCTRL). Three days post-transfection, the transfected-cells were analyzed for SDC1 (black bars) and SDC4 (gray bars) mRNA expression. (B) HCVcc infection was quantified in syndecan-modulated cells by assaying HCV RNA levels by qRT-PCR 3d after exposing cells to Jc1 HCVcc for 4 h at 37°C (MOI = 1) followed by three washes and replacement of media (* = *P*<0.01, ** = *P*<0.001). (C) Huh 7.5.1 cells were transfected with oligonucleotides to knockdown expression of SDC1, SDC4 using a smartpool containing 4 siRNA (siSDC4), SDC4 using siRNA aliquoted individually from siSDC4 (siSDC4-1 to siSDC4-4), CD81 (siCD81) or a siRNA control (siCTRL). Three days post-transfection, transfected cells were infected with Luc-Jc1 HCVcc for 4 h at 37°C. Three days post-infection, infectivity was measured by luciferase reporter activity. HCVcc infection is expressed as a percentage relative to siCTRL-silenced cells (* = *P*<0.01, ** = *P*<0.001). (D) Huh 7.5.1 cells were transfected with oligonucleotides to knockdown expression of SDC4 (siSDC4) or a siRNA control (siCTRL). 24 h post-transfection, cells were transduced with adenoviruses expressing either GFP (Ad-CTRL) as a control, Ad-HA-SDC4-wt or Ad-HA-SDC4-Y180L. Three days post-transduction, transfected and transduced cells were infected with Luc-Jc1 HCVcc for 4 h at 37°C. Three days post-infection, infectivity was measured by luciferase reporter activity. HCVcc infection is expressed as a percentage relative to siCTRL-silenced cells transduced with Ad-CTRL (* = *P*<0.01). (E) HCV pseudoparticle (HCVpp) entry was analyzed by exposure of naïve Huh 7.5.1 cells to sucrose-cushion purified HCVpp (J6) at a MOI of 1 for 4 h at 37°C. Two days after, HCVpp entry was quantified by measuring luciferase activity. Bars represent means±SD of three independent experiments performed in triplicate, of percent change relative to siCTRL. (F) Very-low density lipoprotein (VLDL) at concentrations of 0.5, 5, and 50 µg/mL or PBS as a control were co-incubated with Luc-Jc1 HCVcc (MOI = 1) with naïve Huh 7.5.1 cells for 2 h at 4°C. Following incubation, cells were washed three times with PBS and viral RNA attachment was assessed by qRT-PCR. Bars represent means±SD of three independent experiments performed in triplicate. (* = *P*<0.01, ** = *P*<0.001).

Through defining apoE regions important for HCV entry, we significantly narrowed host binding partners to those that would bind this region and are present on the basolateral surface of hepatocytes. Two proteins meet this criteria; SDC1 and SDC4 [13]. To investigate the role of these factors in HCV entry, we knocked down expression using specific siRNA oligonucleotides to either SDC1, SDC4, or established HCV entry factor CD81, as a positive control. While confirming knockdown by qRT-PCR, we observed an apparent compensation by the alternative syndecan *i.e.* SDC4 increased when SDC1 was silenced and vice versa (Fig. 3A). Knockdown of CD81 also resulted in modest transcriptional stimulation of SDC4 (Fig. 3A). The effect of syndecan modulation of target cells had a clear impact on HCVcc infection, with knockdown of SDC1 only modestly affecting the capacity for infection, whereas knockdown of SDC4 resulted in more than 65% decrease in infection, as quantified by qRT-PCR (Fig. 3B). In order to confirm that viral entry inhibition was not due to off-target silencing, we individually tested the 4 siRNA contributing to the siRNA SDC4 pool previously used. All 4 siRNA targeting SDC4 were able to individually decrease HCV infection (Fig. 3C). Moreover, we also performed a SDC4 complementation assay. First, Huh7.5.1 cells were transfected with a siRNA control (siCTRL) or oligonucleotides targeting SDC4. The following day, these cells were transduced with adenoviral vectors expressing GFP as a control (Ad-CTRL), HA epitope tagged SDC4-wt (Ad-HA-SDC4-wt), or HA epitope tagged SDC4-Y180L (Ad-HA-SDC-Y180L), a mutation important in integrin recycling and therefore potentially interesting in the context of HCV infection [21], [26]. Three days post-transduction, these cells were then challenged with HCVcc Luc-Jc1. As expected, transfections with siCTRL and transductions with Ad-CTRL had no effect on viral entry, whereas the silencing of SDC4 and transduction by Ad-CTRL markedly decreased HCV entry (Fig. 3D). Cells transfected with siSDC4 and transduced with either Ad-HA-SDC4-wt or Ad-HA-SDC4-Y180L partially rescued viral entry (Fig. 3D). The point mutation SDC4-Y180L had no effect on viral entry suggesting that this tyrosine is not involved in HCV entry. This complementation definitively shows that SDC4 is important in HCV infection.

We sought to further define the mechanism of HCV interaction with syndecans by using the HCV pseudoparticle system (HCVpp), which are produced independently of apoE and lipoprotein association [27]. Cells with silenced SDC1 expression were equally capable of HCVpp infection as cells that were either mock transfected or transfected with a nonspecific sequence as a control for silencing (Fig. 3E). However, silencing SDC4 resulted in a modest but significant decrease in the cells’ capacity for HCVpp infection (Fig. 3E). These results, taken together, indicate that SDC4 is a critical factor for apoE-mediated HCV entry, while SDC1 contributes to a lesser degree in mediating HCV infection. Furthermore, SDC4 may have an alternate function that is not dependent on apoE, as evidenced by the results obtained with HCVpp. This may be in part due to HCV E2 glycoprotein binding to HSPG [28].

To further distinguish between HCV usage of SDC1 and SDC4, we took advantage of the fact that SDC1 is the primary receptor for VLDL uptake [15]. Unlike SDC4 knockdown, silencing of SDC1 results in greatly diminished VLDL attachment and uptake into cells [14]. If SDC1 is the primary factor for HCV attachment, HCV and VLDL would act competitively. However, addition of increasing concentrations of VLDL had only a modest effect on competition of HCV attachment to cells (Fig. 3F). While the modest effect may point to a limited use of SDC1, these results are more consistent with SDC1 being excluded as the primary HSPG involved in HCV attachment.

Taken together, our results indicate that HCV infection is mediated by basic residues within HSPG-BD of apoE interacting primarily with SDC4 on the surface of hepatoma cells.

## Discussion

This study clearly demonstrates for the first time that HCV utilizes the HSPG-BD region of apoE to associate with SDC4 and thereby infect hepatoma cells. We further determine using multiple lines of evidence that while HCV uses SDC1 to a limited extent, SDC4 is the primary HSPG used for HCV entry. We have further developed a trans-complementation system to investigate the critical nature of apoE in HCV infection. We have discovered with this system the arresting result that apoE modulation has little effect on the secretion of viral structural proteins, but dramatically correlates with HCV infectivity. A possible explanation for the lack of correlation between the viral protein levels produced and infectivity might be that some non-infectious viral proteins are secreted in exosomes [29]. Our observations are consistent with previous findings that HCV core protein does not correlate with infectivity in buoyant density gradients [4], and that non-infectious HCV can be produced from Huh7 derived cell lines [30]. Strikingly, the infectivity never reached a plateau at the highest apoE expression levels, indicating that apoE is a limiting factor for HCV particle production in the state-of-the-art Huh 7.5.1 cell system. This result is consistent with findings by Long et al. that showed apoE expression renders mouse hepatoma cells capable of infectious HCV production, while noninfectious HCV RNA and core protein is secreted without apoE [31]. Our results here clearly identify the dose-dependent nature of apoE expression and HCV infectivity. HCV appears to have evolved using this liver host factor to target hepatocytes. In fact, more than 90% of circulating apoE is hepatically derived [32].

Utilizing apoE trans-complementation we probed the HSPG-BD of apoE and revealed that elements within this domain are necessary to mediate HCV infection. Deletion of the entire domain or mutation of positively charged residues to alanine resulted in terminating HCV infectivity. The positive charge between each of these residues (R142, K143, R145, and K146) interacts directly with the negatively charged N- and O-sulfo groups of glucosamine sulfate monosaccharides linked to HSPG [33]. This electrostatic interaction may act as an early attachment step to capture HCV on hepatocyte surface, similar to hepatic clearance of lipoproteins [34]. Our results are consistent with previous findings that mutation to alanine of four residues including K143, R145, and K146 diminished infection [10], and expand upon them to show that mutation of only two residues is sufficient to block infection. We further excluded the possibility of the mutations playing a passive role in inhibition of infection, since a peptide consisting of a duplicate of a nine residue sequence from the HSPG-BD successfully out-competed HCV binding and infection. This finding is consistent with previous studies that showed the entire receptor binding region of apoE could inhibit HCV attachment to cells, and it further reveals that a shorter sequence of the HSPG-BD is effective at blocking entry [10].

Since it was clear that the HSPG-BD of apoE was critical for HCV infection, we examined liver-expressed HSPGs for their contribution in mediating infection. Silencing of either SDC1 or SDC4 prior to HCV challenge showed that while knockdown of SDC1 modestly but significantly diminished infection, SDC4 knockdown markedly inhibited infection. Moreover, SDC4-silenced cells transduced with Ad-HA-SDC4-wt or Ad-HA-SDC4-Y180L allowed restoration of viral entry. SDC4 is competent for entry of HCVcc consistent with entry being mediated by apoE, and not through direct binding of the HSPG to HCV glycoprotein E2 [35]. HCVpp lacking apoE showed limited nonspecific interaction with SDC4 emphasizing the role of apoE in HCV attachement. Previous studies have shown that HCVpp lack lipoprotein and apoE associations and are produced in multivesicular bodies [27]; in contrast, HCVcc are dependent upon VLDL components for assembly and release [36]. Indeed, the apoE-derived peptide presented in this study was ineffective at blocking HCVpp infection, consistent with HCVpp infection being independent of apoE association (data not shown).

During the preparation of this manuscript, Shi et al. [37] reported that knockdown of SDC1 inhibits infection with high concentrations of a selected variant. While we observe a limited role of SDC1, our findings clearly indicate through multiple lines of evidence that SDC4 is the primary HSPG involved in HCV infection. Knockdown of SDC4 by Shi et al. [37] did not seem to alter attachment to Huh-7.5 cells, but the authors did not investigate if the knockdown modulated infection. We have observed a putative compensatory mechanism whereby knockdown of SDC1 results in activation of SDC4 expression and vice versa. It is possible that Shi et al. [37] prematurely excluded this compensatory mechanism and the role of SDC4. Their results pointing to the role of SDC1 is in agreement with our observations, however whereas we observe a modest modulation of HCV infection, they observe a robust difference. Possible explanations of this apparent discrepancy may be explained by their use of a high MOI of 10, which may overestimate the role of SDC1.

HCV utilizes aspects of lipoprotein metabolism for propagation, production, and infection. HCV circulates as hybrid complexes with host lipoprotein components. However, HCV also diverges from lipoprotein remnant clearance pathways by using multiple factors including occludin, claudin 1, and CD81 in complex orchestration to mediate entry. The use of SDC4 for apoE mediated HCV entry appears to be one of the first steps that distinguishes HCV infection from lipoprotein remnant clearance, which favors SDC1. This difference between HCV infection and lipoprotein remnant clearance has recently been highlighted by Albecka et al., [12] who showed that LDLR acts more on HCV RNA replication than viral entry. However, soluble LDLR inhibited infection, possibly by competing with HCV-SDC4 binding, since the LDLR binding domain of apoE overlaps the HSPG-BD.

We and others have shown that apoE-specific synthetic peptides are capable of blocking HCV entry [10], [38]. Synthetic anti-lipopolysaccharide peptides that bind to cell surface HSPGs can inhibit infection by a variety of enveloped viruses [39]. Inhibiting apoE-SDC4 interactions represents a novel preventive and therapeutic antiviral strategy that could complement standard-of-care therapies. Indeed, apoE mimetic peptides have already been found to inhibit both viral and bacterial infections. (i.e. herpes simplex type 1 and 2, human immunodeficiency virus, *Pseudomonas aeruginosa, Staphylococcus aureus*) [40], [41]. Inhibition of apoE-SDC4 interactions by using antibodies, peptides, or small molecules could represent a novel strategy in difficult-to-treat patients and prevent infection post liver graft infection, a procedure without preventive strategies and unsatisfactory treatment options [42], [43].
